# A187 PROPHYLACTIC PHAGE TREATMENT ENHANCES SUB-THERAPEUTIC EFFICACY OF BUDESONIDE AGAINST E. COLI DRIVEN COLITIS IN MICE COLONIZED WITH A DEFINED MICROBIOTA

**DOI:** 10.1093/jcag/gwad061.187

**Published:** 2024-02-14

**Authors:** K Jackson, H Galipeau, A Hann, M Bording Jorgensen, B Coombes, Z Hosseinidoust, E Verdu

**Affiliations:** Chemical Engineering, McMaster University Faculty of Engineering, Hamilton, ON, Canada; McMaster University Faculty of Health Sciences, Hamilton, ON, Canada; McMaster University Faculty of Health Sciences, Hamilton, ON, Canada; McMaster University Faculty of Health Sciences, Hamilton, ON, Canada; McMaster University Faculty of Health Sciences, Hamilton, ON, Canada; Chemical Engineering, McMaster University Faculty of Engineering, Hamilton, ON, Canada; McMaster University Faculty of Health Sciences, Hamilton, ON, Canada

## Abstract

**Background:**

Patients with IBD are increasingly experiencing treatment failures on frontline therapies. While corticosteroids are an effective frontline intervention, 16% of patients fail to respond, and 20%–30% show only partial responses. Bacteriophages have recently garnered attention as a potential adjunctive therapy for IBD to target bacterial strains associated with IBD, including adherent-invasive *Escherichia coli* (AIEC).

**Aims:**

Our aim was to determine whether prophylactic bacteriophage therapy could enhance the therapeutic efficacy of a sub-therapeutic dose of budesonide in a gnotobiotic mouse model of *E. coli*-driven colitis.

**Methods:**

Adult germ-free C57BL/6 mice were co-colonized with altered Schaedler-like flora (ASF) plus *E. coli* NRG857c, a Crohn’s disease-associated bacterial isolate. Three weeks later, mice were treated with phage HER259 (1x10^9^ PFU/dose; 0.1% bicarbonate) 3 times/ week or vehicle (PBS with 0.1% bicarbonate) 3 times/ week (n=5/group). Mice were then exposed to low-dose dextran sulfate sodium (2%; DSS) in drinking water for 5 days, followed by 2 days of water. All mice were administered a subtherapeutic dosage of 2ug/day of Budesonide halfway through DSS exposure to endpoint. Mice were monitored daily for weight loss, stool consistency, and fecal occult blood. At sacrifice, colon tissue was collected for histological analysis.

**Results:**

Compared with budesonide treatment alone, prophylactic phage treatment, followed by a sub-therapeutic dose of budesonide, led to reduced stool consistency scores (p ampersand:003C0.001) and reduced presence of occult blood (p ampersand:003C 0.001). No difference in fecal *E. coli* load was observed between groups. At endpoint, the combined phage-budesonide treatment was associated with lower histological scores as compared with mice treated with budesonide alone (p ampersand:003C 0.01).

**Conclusions:**

While underlying mechanisms remain elusive, our results suggest a beneficial effect of prophylactic phage intervention to the subsequent administration of a frontline immunosuppressive compound used to treat IBD. Future work will investigate the mechanisms by which adjunctive phage therapy can enhance existing therapies used to treats colitis, to better inform clinical guidelines.

**Funding Agencies:**

CIHR

